# Atropine-functionalized gold nanoparticles binding to muscarinic receptors after passage across the intestinal epithelium

**DOI:** 10.1098/rsos.220244

**Published:** 2022-10-05

**Authors:** Rebecca Claßen, Ervice Pouokam, Matthias Wickleder, Martin Diener, Annabelle Mattern

**Affiliations:** ^1^ Institute for Veterinary Physiology and Biochemistry, Justus Liebig University Giessen, Frankfurter Strasse 100, 35392 Giessen, Germany; ^2^ Institute of Inorganic Chemistry, University of Cologne, Greinstrasse 6, 50939 Cologne, Germany

**Keywords:** nanoparticles, G-protein coupled receptors, synthesis

## Abstract

Gold nanoparticles have a high potential to be a treatment of diseases by their specific drug delivery properties and multivalent receptor stimulation. For the present project, spherical gold nanoparticles were synthesized and functionalized with the muscarinic receptor antagonist atropine (Au-MUDA-AT NPs). The diameter of the gold core could precisely be controlled by using different synthetic methods and reducing agents resulting in functionalized gold nanoparticles with diameters ranging from 8 to 16 nm. The ability to interact with intestinal muscarinic receptors is size-dependent. When using intestinal chloride secretion induced by the stable acetylcholine derivative, carbachol, as read-out, the strongest inhibition, i.e. the most efficient blockade of muscarinic receptors, was observed with 13 nm sized Au-MUDA-AT NPs. Functional experiments indicate that Au-MUDA-AT NPs with a diameter of 14 nm are able to pass the intestinal mucosa in a time-dependent manner after administration to the intestinal lumen. For example, luminally administered Au-MUDA-AT NPs inhibited contractions of the small intestinal longitudinal muscle layer induced by electrical stimulation of myenteric neurons. A similar inhibition of basolateral epithelial receptors was observed after luminal administration of Au-MUDA-AT NPs when using carbachol-induced chloride secretion across the intestinal epithelium as a test system. Thus, Au-MUDA-AT NPs might be a therapeutic tool for the modulation of intestinal secretion and motility after oral application in the future.

## 1. Introduction

Since 2020, lipid nanoparticles (NPs) gained enormous attention, as this type of NPs is currently being used worldwide as carriers for vaccines against SARS-CoV-2 in the vaccines of Pfizer-BioNTech and Moderna [1,2]. The lipid NPs have been shown to function as protective carriers for modified messenger ribonucleic acid (mRNA) because ribonucleases would otherwise degrade the mRNA inside the body before it could enter the cells and reach the point of action. Therefore, a coating with lipid NPs increases the stability of the mRNA.

In addition, many inorganic nanomaterials gained interest, particularly in biomedical research. With gold NPs (Au NPs) being a prominent example, these structures can function as carriers for an immense variety of biomedical compounds [3–12], and their application reaches from diagnosis [13–16] to therapy [17–20]. Since NPs possess a high surface-to-volume ratio, a large number of functional moieties can be attached. Furthermore, multivalent interactions with cell surface receptors and biomolecules may occur [5,21].

**Figure 1.**
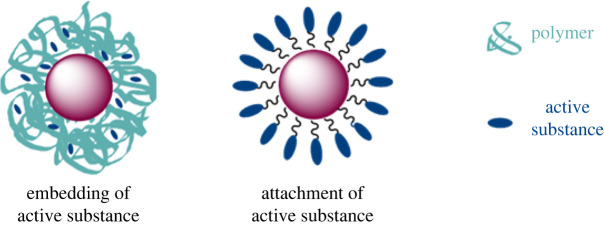
Ways of functionalization: embedding of active substances within the polymer coating (left) or attachment of active substances onto the ligand shell (right) of an Au NP.

One approach in the use of lipid-based NPs is to encage desired drugs in order to transport them through the body. These active substances often possess structures with hydrophobic properties, which makes it necessary to encapsulate them. Furthermore, it is possible to store drugs in a hydrophobic region of a polymer layer-coated Au NP (figure 1, left). These have a hydrophilic outside, which provides sufficient stability of the NPs in aqueous media and ensures an improved transport across biological barriers [22]. Furthermore, a layer-by-layer coating can be applied, in which DNA and RNA may be encapsulated and transported [23]. These modifications may lead to enhanced gene delivery and have been used by Shahbazi *et al.* in their work on CRISPR-functionalized Au NPs [24]. Lee *et al.* used the layer-by-layer coating in their work with silencing RNA, resulting in a gene silencing effect within cancer cells [25].

Another approach is the attachment of the active substances on the ligand periphery (figure 1, right). A linker molecule anchors to the NP surface on one side, while various targeted moieties can be attached to its other side [5,9]. This procedure was performed by Brown *et al*. with the platinum anti-cancer agent oxaliplatin, which was attached via a supramolecular complex to the Au NPs [26]. Although the injected Au NPs showed higher cytotoxicity compared with the free drug, they were effective against colon carcinoma with an IC_50_ of 0.495 nM in comparison with an IC_50_ of 0.775 µM of the free drug oxaliplatin indicating an increase in affinity to the therapeutic target structures [26].

In addition to drugs, also other bioactive compounds, such as neurotransmitters or paracrine mediators, can be attached to the Au NPs. Gasiorek *et al*. showed that histamine bound to Au NPs with the linking unit 11-mercaptoundecanoic acid (MUDA) stimulates histamine receptors on epithelial membranes of a rat already in the subnanomolar range [9]. Furthermore, they observed an enormous potentiation of the receptor activation, as a concentration of 10 pM Au-MUDA-histamine was equieffective to 10 µM native histamine. Even when assuming that each Au-MUDA-histamine particle carried approximately 10 000 molecules of histamine, this corresponds to a strong increase in affinity which was attributed to multivalent interactions [9]. Such a potentiation was also observed in our previous studies on carbachol-functionalized Au NPs [5]. Carbachol (CCh) represents a pharmacologically interesting compound, since it is a stable derivative of acetylcholine, which is not degraded by acetylcholine esterase, the dominant enzyme for cleavage of the native substance, acetylcholine. Thus, muscarinic acetylcholine receptors can be successfully stimulated by CCh-functionalized Au NPs. These might offer potential therapeutic applications, as CCh itself is used therapeutically for glaucoma treatment to reduce intraocular pressure; it has also been used to stimulate the motility of the gastrointestinal tract during post-operative ileus or post-operative retention of urine via stimulation of the motility of the urogenital tract.

Whereas our previous studies were designed to attach neurotransmitters or hormones stimulating distinct G-protein coupled receptors [8], the present experiments focus on the attachment of the receptor antagonist atropine. When absorbed after oral administration, atropine competes with acetylcholine and acts as blocker of muscarinic receptors including M_1_ and M_3_ receptors present at the basolateral membrane of the epithelium involved in the activation of epithelial secretion [27] or M_2_ and M_3_ receptors involved in the stimulation of gastrointestinal motility [28]. Thus, there is interest in whether Au NPs can function as carriers for atropine and whether they still cross the epithelial barrier to act on the basolateral side of the epithelium or the smooth muscle layers below the mucosa.

Furthermore, atropine or other anticholinergic drugs such as hyoscine butylbromide are currently used to treat spasms in the gastrointestinal tract [29]. Thus, atropine-functionalized Au NPs (Au-MUDA-AT NPs) could have potential therapeutic applications, as atropine or related anticholinergic drugs are widely used in medicine, e.g. to treat bradycardia, as premedicamentation during narcosis, or to overcome intestinal spasms [30]. Nanoparticles are not only passive drug vehicles. In particular, their effects are generally regarded to be strongly dependent on their physico-chemical properties such as e.g. shape, size, chemical composition [31,32]. Therefore, Au-MUDA-AT NPs of different sizes were synthesized and their effects on the intestinal tissue were investigated.

## 2. Results and discussion

### 2.1. Nanoparticle syntheses

**Figure 2.**
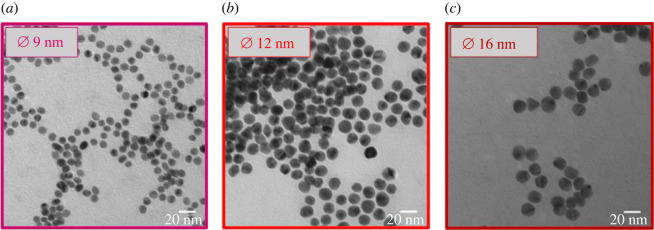
TEM images of Au-MUDA NPs with Ø 9 nm (*a*), Au-citrate NPs with Ø 12 nm (*b*) and Ø 16 nm (*c*).

Monodisperse, spherical Au NPs were synthesized in order to use them as starting particles for a further biofunctionalization with atropine. The citrate reduction method was employed, as described in our previous publications [5,8]. Moreover, by reacting different ratios of HAuCl_4_ × 3 H_2_O and sodium citrate various sizes of Au NPs were selectively synthesized with individual diameters ranging from 11 nm (HAuCl_4_ × 3 H_2_O: sodium citrate × 2 H_2_O ratio of 1.0 : 15.0) to 16 nm (figure 2*c*; HAuCl_4_ × 3 H_2_O: sodium citrate × 2 H_2_O ratio of 1.0 : 4.0). It shows that the higher the percentage of citrate in the reaction mixture compared with tetracholoauric acid, the smaller the obtained NP diameter.

Furthermore, smaller Au NPs (Au-MUDA 8 nm, Au-MUDA 9 nm (figure 2*a*), Au-MUDA 10 nm) were synthesized according to our published procedure [8]. Here, MUDA was used as the stabilizing ligand and ^t^Bu-amine borane complex as a stronger reducing agent [33], resulting in slightly smaller Au NPs as synthesized using the citrate reduction method [34]. All Au NP dispersions were obtained in H_2_O and analytical characterizations revealed stable, spherical and monodisperse Au NP dispersions.

### 2.2. Ligand synthesis and nanoparticle functionalization

**Figure 3.**
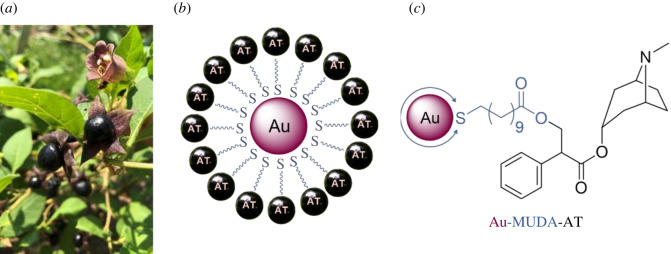
Image of deadly nightshade *Atropa belladonna* (photo: Annabelle Mattern) (*a*), scheme (*b*) and structure of atropine-functionalized Au NPs (*c*).

**Scheme 1.**
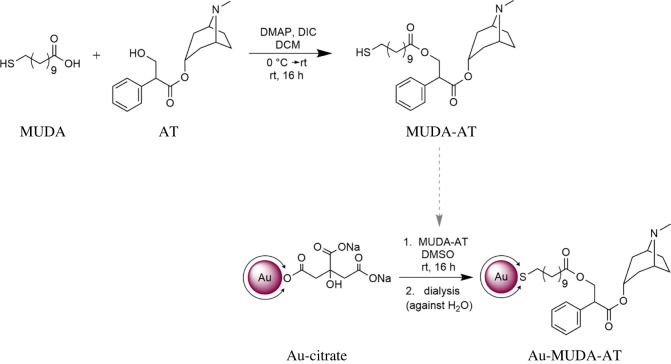
Synthesis of MUDA-AT in a Steglich esterification (top) and NP functionalization in a ligand exchange reaction with MUDA-AT (bottom).

Hereinafter, the biofunctionalization of Au NPs with atropine is described. Atropine itself is found as the poison of the deadly nightshade *Atropa belladonna* (figure 3*a*) in nature. The chemical structure of this compound already contains an ester function (figure 3*c*, black). In order to immobilize it onto the Au NPs, atropine (purchased in pure form from Sigma Aldrich) was linked with MUDA as a thiol-containing bifunctional linker. An ester bond was formed with atropine's free hydroxyl group and MUDA's acid moiety (figure 3*c*). The esterification was performed under mild reaction conditions under inert atmosphere according to a modified procedure described by Neises & Steglich [35]; scheme 1, top displays the ligand synthesis. MUDA was dissolved in anhydrous dichloromethane (DCM) and catalytic amounts of 4-(dimethylamino)pyridine (DMAP) (0.1 eq.) were added. The coupling reagent diisopropyl carbodiimide (DIC) was added to the cooled solution and then the reaction mixture was treated with atropine. In particular, sterically demanding and acid-labile reactants may undergo reactions under mild conditions at room temperature in this approach. Remaining acid and other side products were removed in the subsequent aqueous work-up. The crude product was purified over a silica plug. Traces of the side product N,N′-diisopropylurea (DIU) remained and could not be removed completely during various work-up steps. However, these were tolerated, as the desired biofunctional ligand possesses a thiol moiety, which binds more favourably to the NPs.

The ligand was analysed using NMR and IR spectroscopy. The ^1^H-NMR spectrum shows all expected proton resonances. Characteristic resonances in the range from 1.63 to 1.10 ppm correspond to the long alkyl chain of the thiol spacer MUDA. Furthermore, aromatic proton signals between 7.30 and 7.18 ppm can be assigned to the phenyl unit of atropine and suggest a successful ligand synthesis. IR measurements were performed to verify the existing binding types within the product. The spectrum of MUDA-AT reveals distinct *ν*_C–H_ resonances in the range of 3000–2800 cm^−1^, which are significantly stronger than in the spectrum of AT and thus indicate the long alkyl chain of MUDA. Furthermore, MUDA-AT shows an intense *ν*_C–S_ vibration at 629 cm^−1^, suggesting the existence of a thiol.

With the synthesized ligand MUDA-AT, functionalization of the Au NPs was performed in a ligand exchange reaction (scheme 1, bottom). For this purpose, an Au NP dispersion of the preferred size was degassed with argon. Then, MUDA-AT was dissolved in DMSO and slowly added in a large excess under vigorous stirring at room temperature. The ligand was added in a dissolved form in order to ensure rapid and efficient homogenization. Furthermore, agglomeration of the Au NPs upon the addition of bulk material is prevented.

**Figure 4.**
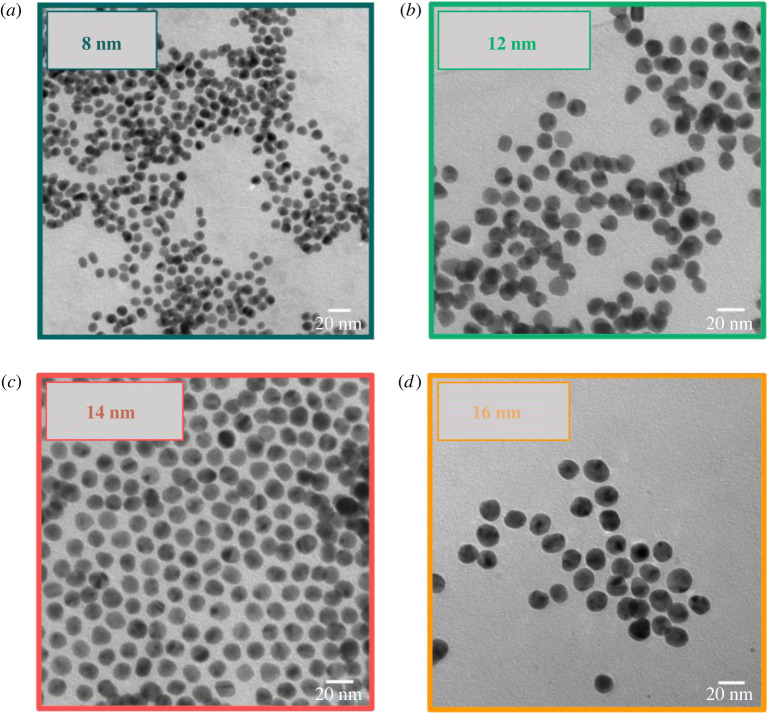
TEM images of Au-MUDA-AT NPs with a size of Ø 8 nm (*a*), Ø 12 nm (*b*), Ø 14 nm (*c*) and Ø 16 nm (*d*).

**Figure 5.**
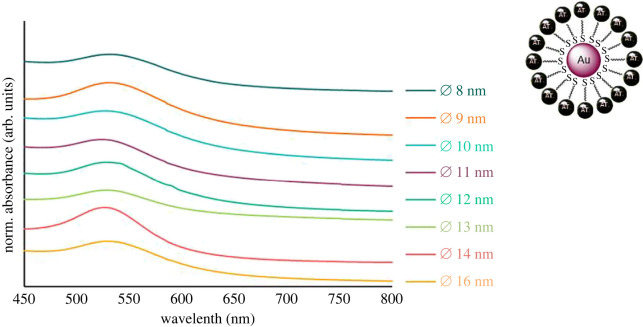
UV/Vis spectra of Au-MUDA-AT NPs.

**Figure 6.**
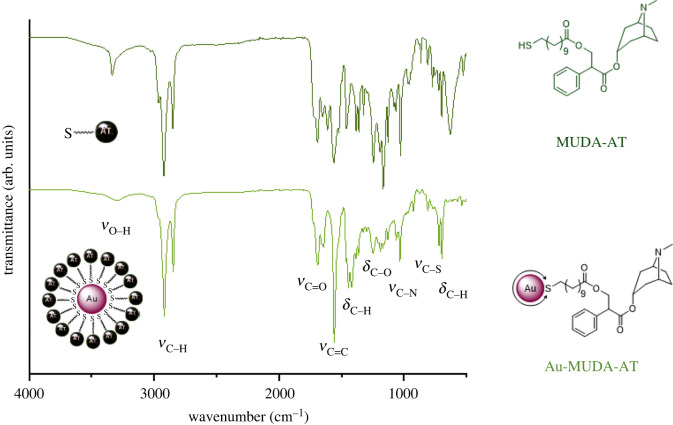
IR spectra of the free ligand MUDA-AT (top) and Au-MUDA-AT NPs (bottom).

The obtained pink and stable Au NP dispersion was purified via dialysis in order to remove free ligands and further side products. All NP samples were characterized with TEM, DLS and UV/Vis. Furthermore, the organic surface structure was confirmed using NMR and IR spectroscopy. TEM images of Au-MUDA-AT NPs show predominantly spherical, monodisperse NPs (figure 4). No remarkable change in the morphology of the NPs occurred during their functionalization with MUDA-AT and they furthermore possess a small size distribution in their expected diameters (table 1) between Ø 8 nm (figure 4*a*) and Ø 16 nm (figure 4*d*). Moreover, Au-MUDA-AT NPs with Ø 14 nm (figure 4*c*) even arranged themselves hexagonally on the TEM grid, indicating a particularly high monodispersity. No agglomeration was observed in all approaches; thus, MUDA-AT reveals to be a suitable ligand to sufficiently stabilize Au NPs. The *d*_hydr_ values all revealed to be slightly larger after the functionalization with MUDA-AT (table 1), which is expected, since a new sterically high demanding ligand is found on the Au NP surface. The UV/Vis spectra of all Au-MUDA-AT NPs (figure 5) show a distinct plasmon resonance with an absorption maximum *λ*_max_ each in the range of around 530 nm. Since only one relatively narrow absorption band with a noticeable rise can be observed, a stable dispersion with monodisperse NPs can be assumed. The ^1^H-NMR spectra of Au-MUDA-AT NPs show all desired proton resonances which are also found in the spectrum of the synthesized ligand MUDA-AT. Characteristic proton resonances between 1.74 and 1.15 ppm correspond to the long alkyl chain of MUDA-AT. Furthermore, aromatic proton resonances in the range from 7.49 to 7.19 ppm are assigned to the phenyl unit present in atropine. Additionally, the IR spectrum of the Au-MUDA-AT NP sample (figure 6, bottom) was compared with the one of the free ligand MUDA-AT (figure 6, top) and these show unambiguous similarities. Again, the intensive *ν*_C–H_ absorptions in the range of 2900 cm^−1^ confirm the presence of the long chain MUDA. Moreover, both *ν*_C=O_ absorptions between 1730 and 1650 cm^−1^ and the *δ*_C–O_ vibrations around 1200 cm^−1^ reveal the ester groups on the organic ligand sphere of the NP samples. Even the entire fingerprint areas resemble one another very closely.

**Table 1.** Properties of Au-MUDA-AT NP samples.

size	8 nm	9 nm	10 nm	11 nm	12 nm	13 nm	14 nm	16 nm
sample	Au-MUDA-AT							
*d*_TEM_/nm	8.1 ± 0.07	9.1 ± 0.7	9.7 ± 0.9	11.2 ± 1.2	12.0 ± 1.0	12.9 ± 1.0	14.0 ± 1.0	15.9 ± 1.1
*d*_hydr_/nm	29 ± 8	15 ± 6	33 ± 4	31 ± 5	112 ± 59	105 ± 42	31 ± 13	53 ± 19
*λ*_max_/nm	530	530	530	523	529	528	527	528

In summary, these data demonstrate the successful functionalization of Au NPs with the biogenic substance atropine for different selected Au NP sizes.

### 2.3. Biological actions of the atropine-functionalized gold nanoparticles

#### 2.3.1. Epithelial uptake of Au-MUDA-AT NPs, Ø 14 nm

##### 2.3.1.1. Ussing chamber experiments

Crossing the intestinal barrier is a major obstacle for many drugs, limiting their therapeutic use. For a future nanoparticle-based oral drug delivery, which might increase the absorption of drugs, it is necessary that Au-MUDA-AT NPs can overcome the intestinal barrier to reach the muscarinic receptors located on the basolateral cell side of the epithelium [36].

**Figure 7.**
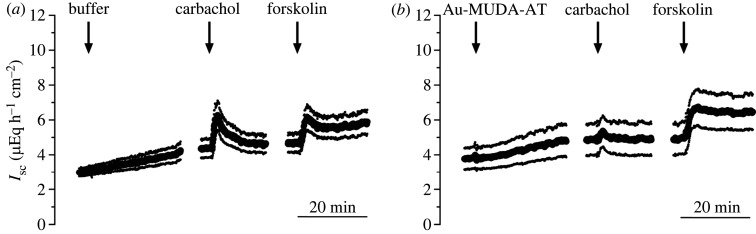
Time course of Ussing chamber experiments. Equivalent volumes of buffer (*a*) or Au-MUDA-AT NPs (*b*; Ø 14 nm, 2 × 10^−10^ mol l^−1^) were applied at the mucosal side of the jejunum. After 0.5 h incubation, carbachol (5 × 10^−5^ mol l^−1^ at the serosal side) was administered. Forskolin (9 × 10^−6^ mol l^−1^ at the serosal and mucosal side) served as viability control at the end of each experiment. Data are means (thick lines) ± s.e.m. (thin lines), *n* = 7–8. For statistics, figure 8.

**Figure 8.**
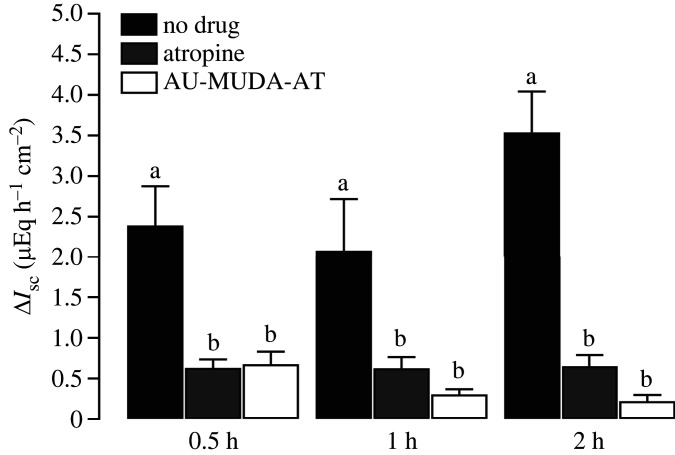
Change of *I*_sc_ induced by carbachol (5 × 10^−5^ mol l^−1^ at the serosal side) after pre-treatment with buffer (no drug, black bars), atropine (2 × 10^−8^ mol l^−1^, grey bars) or Au-MUDA-AT NPs (Ø 14 nm, 2 × 10^−10^ mol l^−1^, white bars) at the mucosal side of the jejunum. Incubation periods for the different drugs were 0.5, 1 and 2 h, respectively. Letters indicate statistically homogeneous groups within the same incubation period, *p* < 0.05. Data were analysed using one-way ANOVA followed by Tukey *post hoc* test and are shown as means (bars) + s.e.m. (lines), *n* = 5–8.

The stable acetylcholine derivative carbachol (CCh) is well known to stimulate muscarinic receptors and induce intestinal chloride secretion which can be measured as increase of short-circuit current (*I*_sc_) in Ussing chamber experiments [37]. This CCh-induced increase of *I*_sc_ is inhibited by atropine [38]. The ability of Au-MUDA-AT NPs to diminish the CCh-induced effects in the intestine after mucosal application was measured in Ussing chamber experiments and compared with the response obtained with native atropine. For this purpose, segments of rat jejunum were incubated with Au-MUDA-AT NPs (Ø 14 nm, 2 × 10^−10^ mol l^−1^), atropine (2 × 10^−8^ mol l^−1^) or equivalent volumes of buffer on the mucosal (i.e. the luminal) side of the tissue for different periods of time (0.5, 1 or 2 h). This was followed by administration of CCh (5 × 10^−5^ mol l^−1^) at the serosal side to stimulate basolaterally located muscarinic receptors at the epithelial cells. The CCh-induced current, measured as increase in *I*_sc_ above the baseline just prior to administration of CCh (Δ*I*_sc_), was registered (figures 7 and 8). Tissues pre-treated with Au-MUDA-AT NPs or atropine showed a significantly reduced response (*p* < 0.05) to CCh when compared with time-dependent control tissues, which were not treated with cholinergic antagonists, but received the buffer for NP administration, only.

Furthermore, the inhibition of the CCh response by Au-MUDA-AT NPs was intensified, when incubation time was increased from 0.5 to 1 or 2 h; a phenomenon, which was not observed for native atropine. This suggests that native atropine is absorbed quicker by the epithelium than atropine bound to Au NPs, as native atropine shows its maximum inhibitory effect already at an earlier time point. Apparently, the Au-MUDA-AT NPs crossed the intestinal epithelium in a time-dependent manner: the longer the incubation period lasted, the more NPs reached the basolateral side of the epithelium. At the end of each experiment, forskolin (9 × 10^−6^ mol l^−1^ at the serosal and mucosal side), a cAMP-dependent activator of chloride secretion [39], was administered. No significant differences in the change of *I*_sc_ (Δ*I*_sc_) were observed in the forskolin response between all groups, indicating that Au-MUDA-AT NPs did not unspecifically affect the secretory capability of the epithelium. In addition, tissue conductance (*G*_t_) was not altered after drug application, indicating that neither atropine nor Au-MUDA-AT NPs affected the viability of the intestinal epithelium (data not shown).

##### 2.3.1.2. Organ bath experiments and electrical field stimulation

**Figure 9.**
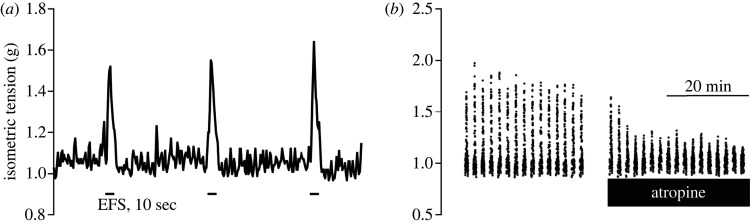
Example of isometric tension measurement during electrical field stimulation of the inverted jejunum. Lines indicate application of electrical stimuli for 10 s (*a*). Decrease of EFS-induced contractions after atropine application (2 × 10^−5^ mol l^−1^ at the mucosal side) (*b*). Contraction peaks occurred simultaneously to EFS application. Data in *b* are means (black dots) ± s.e.m. (grey dots), *n* = 11.

As the modulation of intestinal motility would be an interesting therapeutic application, e.g. for the treatment of intestinal spasms, organ bath experiments were performed to measure the change of isometric force of the tunica muscularis after Au-MUDA-AT NPs administration. First, the general ability of Au-MUDA-AT NPs to modulate jejunal contractility was shown after serosal application. To do so, intestinal segments in their natural geometry (i.e. not inverted as done in later experiments, see below) with the tunica muscularis in direct contact to the organ bath solution, were used, so that the Au-MUDA-AT NPs administered to the bath solution had direct access to the muscarinic receptors within the muscle layer. Au-MUDA-AT NPs (Ø 14 nm) at a concentration of 5 × 10^−12^ mol l^−1^ led to a significant decrease (*p* < 0.05) of CCh-induced (5 × 10^−7^ mol l^−1^ at the serosal side) isometric force by approximately two-thirds in comparison with the untreated group (0.52 ± 0.13 g versus 1.66 ± 0.38 g; mean ± s.e.m., *n* = 8; data not shown). Second, the modulation of contractility after intestinal uptake of the NPs was investigated. For this purpose, segments of the jejunum were inverted, so that the mucosa faced the outer surface of the resulting tube and atropine or Au-MUDA-AT NPs, which were administered to the organ bath, first had to pass the mucosa before they could reach muscarinic receptors on the smooth muscle cells of the tunica muscularis. These inverted jejunal segments were electrically stimulated by electric field stimulation (current pulses of 250 mA, pulse duration of 1 ms, frequency 10 Hz, train duration 10 s), which was repeated every 2 min. These pulses evoke an increase in isometric force due to the release of excitatory neurotransmitters from myenteric neurons innervating the smooth muscle cells (figure 9*a*). These so-called on-contractions, which occur simultaneously to electrical field stimulation (EFS), are described to be atropine-sensitive [40]. Indeed, atropine (2 × 10^−5^ mol l^−1^ at the mucosal side) led to a reduction of contraction strength under EFS approximately more than 60% after a 30 min incubation period (figure 9*b*, table 2).

**Table 2.** Change of isometric force under EFS after treatment with atropine (2 × 10^−5^ mol l^−1^ at the mucosal side), Au-MUDA-AT NPs (Ø 14 nm, 2 × 10^−10^ mol l^−1^ at the mucosal side) or no drug (equivalent volumes of buffer at the mucosal side). Contractions prior to drug application (pre-drug period) were averaged and set to 100%. The following contractions were averaged over different time periods and compared with the pre-drug period. Letters are statistically homogeneous groups within one treatment group, *p* < 0.05. Data were analysed using one-way ANOVA followed by Tukey *post hoc* test and represent means ± s.e.m.

treatment	period	increase of force (g)	% of initial force	*n*
no drug	pre-drug period	0.80 ± 0.07^a^	100.00 ± 2.63^a^	8
	0–30 min	0.85 ± 0.07^a^	128.11 ± 6.13^b^	
	30–60 min	0.82 ± 0.07^a^	115.03 ± 5.06^a,b^	
	60–90 min	0.83 ± 0.08^a^	117.66 ± 5.17^a,b^	
	90–120 min	0.80 ± 0.08^a^	113.40 ± 6.15^a,b^	
atropine	pre-drug period	0.81 ± 0.04^a^	100.00 ± 1.52^a^	11
	0–10 min	0.47 ± 0.05^b^	62.83 ± 4.18^b^	
	10–20 min	0.27 ± 0.03^b,c^	35.38 ± 2.24^c^	
	20–30 min	0.26 ± 0.03^c^	33.79 ± 2.60^c^	
Au-MUDA-AT	pre-drug period	1.06 ± 0.07^a^	100.00 ± 1.65^a^	7
	0–30 min	1.01 ± 0.07^a,b^	91.87 ± 2.52^a^	
	30–60 min	0.95 ± 0.08^a,b^	79.78 ± 3.08^b^	
	60–90 min	0.88 ± 0.07^a,b^	76.19 ± 3.52^b^	
	90–120 min	0.76 ± 0.07^b^	60.20 ± 3.65^c^	

**Figure 10.**
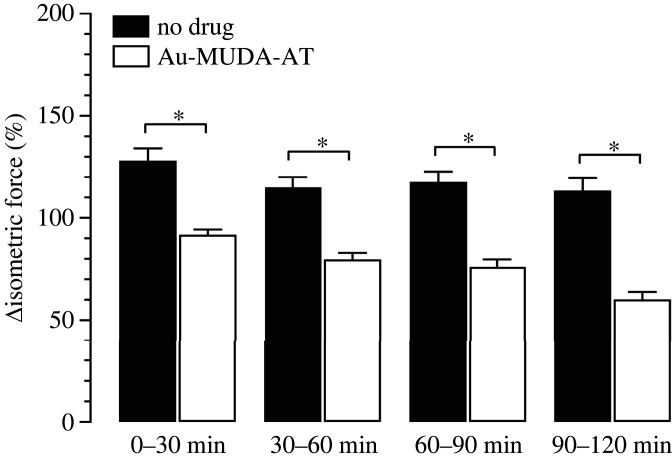
Change of isometric force under electrical field stimulation after application of Au-MUDA-AT NPs (Ø 14 nm, 2 × 10^−10^ mol l^−1^ at the mucosal side, white bars) or equivalent volumes of buffer (black bars). Values were averaged over 30 min and set in relation to pre-drug period (set to 100%). For absolute values, table 2. Data are means (bars) + s.e.m. (lines), *n* = 7–8. * *p* < 0.05, Student's *t*-test followed by Mann–Whitney *U*-test.

The mucosal application of Au-MUDA-AT NPs required a higher concentration of 2 × 10^−10^ mol l^−1^ to be effective. In this concentration, the atropine-functionalized NPs inhibited the EFS-induced contractions significantly in a time-dependent manner. For statistical analysis, the EFS-induced increases of isometric force prior to drug application for each muscle segment were averaged over 30 min (i.e. the average of 15 on-contractions during the untreated control period (pre-drug period) was calculated for each jejunal segment) and set as 100%. All following changes of isometric force were averaged in periods of 10 min (atropine) or 30 min (no drugs, Au-MUDA-AT NPs) and compared in % with this reference value calculated for the pre-drug period. In the time-dependent control series, i.e. without administration of any drugs, these EFS-induced contractions did not decrease but had in contrast the tendency to increase slightly over time (table 2). When atropine (2 × 10^−5^ mol l^−1^ at the mucosal side) was administered, a fast decrease of the EFS-induced contractions was observed, which reached a stable plateau already 10 min after administration of this muscarinic antagonist (table 2). A significant decrease in the EFS-induced contractions was also observed after administration of Au-MUDA-AT NPs (2 × 10^−10^ mol l^−1^ at the mucosal side), which developed, however, more slowly in comparison with native atropine (figure 10, table 2). This indicates that Au-MUDA-AT NPs cross the intestinal epithelium in a time-dependent manner as already suggested from the data obtained in the Ussing chamber experiments.

Taken together, the findings from the uptake studies indicate that Au-MUDA-AT NPs (Ø 14 nm) can cross the intestinal barrier in a time-dependent manner and bind to muscarinic receptors on the basolateral side of the enterocytes. Further studies are necessary to understand how Au-MUDA-AT NPs overcome the intestinal epithelium. Generally, there are different strategies to cross membranes including endocytosis, which plays a major role as cell entry mechanism for NPs [41]. By binding specific ligands to the NPs' surface, the NPs can actively target specific cell receptors and enter the cells via receptor-mediated endocytosis (RME) [42]. For example, insulin-loaded polymeric NPs whose surface was coated with the protein-ligand transferrin were internalized after specific receptor binding and induced hypoglycaemia in rats [43]. Furthermore, Au NPs whose surface was not loaded with a specific ligand were endocytosed via RME after non-specific serum protein adsorption onto the nanoparticles' surface [44]. Another strategy for crossing the intestinal barrier is paracellular trafficking, e.g. after tight junction opening [45,46]. As there is a great diversity in the physico-chemical properties of NPs in general (e.g. composition, stabilizing agent, surface modification, surface charge, size, shape, etc.) which influence their cellular uptake [47–49], the specific cell entry of an NP has to be evaluated individually. Therefore, future studies are necessary to investigate how Au-MUDA-AT NPs exactly cross the intestinal barrier. For a possible *in vivo* application, also environmental factors as the acidic pH in the stomach, the enzymes of the digestive tract or the intestinal mucus will determine the uptake of the NPs and have to be considered during synthesis. Additionally, the NPs' biodistribution in the organism after crossing the intestinal barrier has to be evaluated and can be affected by the chemistry of the NPs [50,51].

In 2020, Enea *et al*. performed *in vitro* studies with Caco2-cells and also 11-MUDA acid-coated gold nanospheres [52]. By contrast to our results, neither 15 nm nor 60 nm sized NPs could cross the cells but only accumulated intracellularly. This indicates that the intestinal crossing of our 14 nm sized Au-MUDA-AT NPs occurred due to the slight size difference or the surface functionalizsation. Apparently, Au-MUDA NPs induce only low cytotoxic effects [52,53], even high-dose oral application of Au NPs over 14 days did not cause severe toxicity [54], offering a promising perspective for a possible future oral drug delivery of Au-MUDA-AT NPs. Not only the local treatment of intestinal diseases, as intestinal spasms, is of therapeutic interest, also systemic delivery of different functionalized Au NPs would be beneficial. Thus, the results of our uptake studies might help in the development of novel nanoparticle-based therapies.

### 2.4. Impact of Au NP core sizes

**Figure 11.**
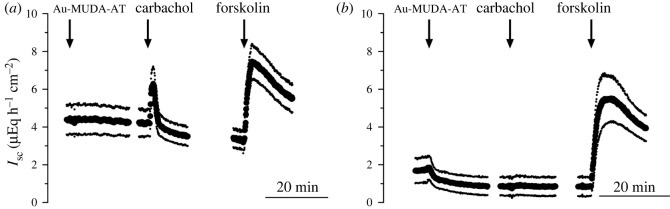
Response to carbachol (5 × 10^−5^ mol l^−1^ at the serosal side) after incubation with Au-MUDA-AT NPs (2 × 10^−10^ mol l^−1^ at the serosal side) with 8 nm diameter (*a*) and 13 nm diameter (*b*). Forskolin (9 × 10^−6^ mol l^−1^ at the serosal and mucosal side) was applied as viability control at the end of each experiment. Data are means (thick line) ± s.e.m. (thin lines), *n* = 8–9. For statistical analysis, figure 12.

**Figure 12.**
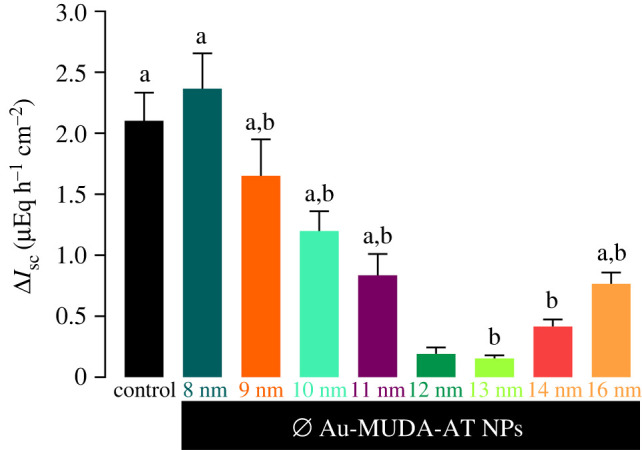
CCh-induced (5 × 10^−5^ mol l^−1^ at the serosal side) Δ*I*_sc_ after 20 min incubation with Au-MUDA-AT NPs (2 × 10^−10^ mol l^−1^ at the serosal side; *n* = 8–9) with different diameters in comparison with the control group (grouped controls from all experimental series; *n* = 73). Forskolin (9 × 10^−6^ mol l^−1^ at the serosal and mucosal side) served as viability control at the end of each experiment. Data are presented as means (bars) + s.e.m. (thin lines). Letters indicate statistical homogeneous groups, *p* < 0.05. One-way ANOVA followed by Tukey *post hoc* test was performed as statistical analysis.

As the effects of NPs are strongly dependent from their properties [31,32], the effects of Au-MUDA-AT NPs with different diameters (8–16 nm) were tested in Ussing chamber experiments (figure 11). After 20 min of Au-MUDA-AT NPs incubation (2 × 10^−10^ mol l^−1^) at the serosal side of the jejunum, CCh (5 × 10^−5^ mol l^−1^ at the serosal side) was administered. Again, forskolin (9 × 10^−6^ mol l^−1^ at the serosal and mucosal side) was administered as viability control at the end of each experiment (figure 11) [39]. For a better stabilization of *I*_sc_ prior to drug application, tissues were pre-treated with the non-steroidal anti-inflammatory drug indomethacin (10^−6^ mol l^−1^ at the mucosal and serosal side). Indomethacin inhibits stimulation of epithelial secretion via release of prostaglandins [55]. Au-MUDA-AT NPs with a diameter of 13 nm showed the strongest inhibition of the carbachol-induced *I*_sc_ with approximately more than 75% compared with control tissues. The strength of inhibition decreased steadily with smaller and larger diameters of Au-MUDA-AT NPs (figure 12). In none of the experimental series, the secretory response evoked by the cAMP-dependent secretagogue forskolin (9 × 10^−6^ mol l^−1^ at the serosal and mucosal side) was significantly lower than in the untreated control group, clearly excluding unspecific inhibition of the transporters involved in anion secretion by the NPs. The tissue conductance was not altered after NPs treatment, too (data not shown).

These results prove that the effects of Au-MUDA-AT NPs are strongly size-dependent with 13 nm sized Au NPs showing the strongest effects. This might be explained by the different curvature of different-sized NPs [56]. The given curvature influences ligand–receptor interactions as a specific angle or arrangement of the ligands on the NP surface can promote or hinder the formation of multiple receptor–ligand complexes at the same time. Other studies have also focused on the relation between the effects of the NPs and their size, but mainly larger size distributions were investigated. The focus on this small size range from 8 to 16 nm shows that fine-tuning of the NPs' properties is very important to reach the most promising effects. As Jiang *et al.* have described in their study in 2008, not only the surface or the functionalization determines the NPs' interaction with cells but also the NPs and their specific properties themselves [32]. These are important findings that must be regarded when NPs are used in therapeutic application.

## 3. Conclusion

In conclusion, the successful synthesis and the proof of biological effectiveness of atropine-functionalized gold nanoparticles with a narrow size distribution were shown. Functional studies demonstrated that Au-MUDA-AT NPs can cross the intestinal barrier and modulate the secretion and motility of the gut. The size-dependent interaction with muscarinic receptors emphasized how important the nanoparticles' properties are. Our findings suggest a possible use of functionalized gold nanoparticles as a future treatment of intestinal diseases after oral application.

## 4. Experimental section

### 4.1. Chemical, materials and laboratory techniques

All chemicals were purchased from *Acros Organics*, *Alfa Aesar*, *Carl Roth*, *Fisher Scientific*, *Fluka*, *Merck*, *Santa Cruz Biotechnology*, *Sigma Aldrich* or *TCI* and used without further purification. Organic solvents were distilled before use or purchased in an anhydrous state and stored over molecular sieves. Chromatographic purifications were performed using *Merck* silica gel 60 (0.040–0.063 mm). Thin layer chromatography (TLC) was performed on *Merck* aluminium-backed plates with silica gel and fluorescent indicator (254 nm). For indication, UV light (*λ* = 254 nm/365 nm) was used.

Reactions were performed under inert conditions (argon atmosphere 99.9999%, *Air Products*) using standard Schlenk line techniques with oven-dried glassware unless stated otherwise. All water-based (nanoparticle) syntheses were performed in demineralized water or *arium* water. All glass vessels were washed with aqua regia and demineralized water prior to use. For dialysis of the NP solutions, membranes of regenerated cellulose ‘*ZelluTrans*’ with different pore sizes (MWCO 6000 and MWCO 12 000) were used. These were purchased from *Carl Roth GmbH*. The dialysis membrane was immersed in the dialysing solvent for 30 min prior to use. All dialyses were performed at room temperature.

### 4.2. Analytical methods

^1^H-NMR and ^13^C-NMR spectra were recorded on *Bruker Avance 400 MHz (AV 400)*, *Bruker Avance II 300 MHz (AV 300)* and *Bruker Avance II+ 600 MHz (AV II 600)* spectrometers at the Institute of Organic Chemistry, University of Cologne. All measurements were performed at room temperature. Chemical shifts are given in ppm relative to respective solvent peaks. ^1^H-NMR data are reported as follows: chemical shifts (multiplicity [ppm], classification). Multiplicity is recorded as s = singlet, d = doublet, t = triplet, q = quartet, m = multiplet. All spectra were displayed with the software MestReNova. ^1^H-NMR spectra of all Au NP dispersions were recorded. The Au NP dispersion (4–5 ml) was dried *in vacuo* and subsequently dissolved in D_2_O in order to prepare the samples. To ensure stable dispersions during the measurements, a base (usually NEt_3_) in D_2_O was added. The measured NMR spectra were then compared with the spectra of the initial Au NP dispersions as well as the synthesized ligands. Impurities of D_2_O and NEt_3_ originate from the sample preparation. Mass spectra were recorded on an ESI mass spectrometer (spectrometer (*micrOTOF*) from *Bruker Daltonics (Bremen, Germany)*) at the Justus Liebig University Giessen or on an ESI mass spectrometer of *Agilent Technologies*, model *LC/MSD Vl*, at the research group of Prof. Berkessel at the University of Cologne. IR measurements were performed on a *Perkin Elmer* FTIR-ATR (*UATR TWO*) at room temperature with a maximum resolution of 1 cm^−1^. Absorption bands are given in cm^−1^. TEM images were taken on a Zeiss *LEO 912* (300 kV, LaB_6_-cathode) equipped with a GATAN digital camera. For sample preparation, a 10 µl NP solution was placed on a carbon-coated copper grid. Determination of the average particle size and standard deviation was achieved by measuring 200 individual particles using *Image J Fiji*. Dynamic light scattering (DLS) measurements were performed on a *NanoZS* from *Malvern.* UV/Vis spectra were recorded with an *UV-1600PC* spectrophotometer from *VWR*. Elemental analyses for C, H, N and S were acquired by Mr Dirk Pullem on a *Eurolab EA Elemental Analyzer* at the University of Cologne.

### 4.3. Ligand synthesis

#### 4.3.1. Synthesis of MUDA-AT (according to a method by Neises & Steglich [35])

MUDA (150 mg, 0.69 mmol, 1.0 eq.) was dissolved in anhydrous DCM (10 ml). DMAP (9 mg, 0.07 mmol, 0.1 eq.) was added under argon counter flow and stirred at 0°C. DIC (150 µl, 0.97 mmol 1.4 eq.) was added quickly and stirred for 5 min at 0°C. Then, AT (200 mg, 0.69 mmol, 1.0 eq.) was added and stirred for 15 min at 0°C. Afterward, the reaction mixture was stirred at room temperature for 16 h under argon atmosphere. During the work-up process, the reaction mixture was filtered and the solvent was evaporated. The residue was redissolved in DCM and washed with diluted HCl (2 × 10 ml) and with saturated NaHCO_3_ solution (10 ml). The combined organic layers were dried over Na_2_SO_4_, filtered and the solvent was evaporated. The crude product was purified over a silica plug (eluted with DCM). The product could be obtained as a colourless solid in a good overall yield (283 mg, 84%) and was stored at 4°C in the dark.

**^1^H-NMR** (400 MHz, CDCl_3_): *δ*/ppm = 7.30–7.18 (m, 5 H, C*H*), 5.23 (s, 1 H, C*H*), 4.99–4.93 (m, 1 H, C*H*), 4.53 (t, *J* = 9.3 Hz, 1 H, C*H*), 4.23 (t, *J* = 6.7 Hz, 1 H, C*H*), 2.60 (t, *J* = 6.7 Hz, 2 H, C*H*_2_), 2.27 (s, 3 H, C*H*_3_), 2.33–2.12 (m, 4 H, C*H*_2_), 1.85–1.67 (m, 4 H, C*H*_2_), 1.63–1.41 (m, 6 H, C*H*_2_), 1.36–1.10 (m, 10 H, C*H*_2_); **^13^C{^1^H} NMR** (100 MHz, CDCl_3_): *δ*/ppm = 172.2, 169.5, 133.9, 127.9, 127.8, 127.0, 126.6, 66.5, 63.5, 58.6, 50.0, 35.6, 35.2, 33.9, 31.86, 28.6, 28.4, 28.2, 27.8, 27.5, 27.3, 24.9, 24.6; **IR** (ATR): *ν*/cm^−1^ = 3335, 2921, 2842 (*ν*_C–H_), 1730 (*ν*_C=O_), 1695 (*ν*_C=O_), 1558, 1542 (*ν*_C=C_), 1465, 1390, 1360 (*δ*_C–H_), 1250 (*δ*_C–O_), 1166 (*δ*_C–O_), 1029 (*ν*_C–N_), 773 (*δ*_C–H_), 729 (*ν*_C–S_), 700 (*δ*_C–H_), 632 (*δ*_C–H_); **ESI-MS (*m/z*):** [M-H]^−^ = 488.23 (calcd: [M-H]^−^ = 488.28); **Elemental Analysis:** anal. calcd for (C_28_H_43_NO_4_S)(C_7_H_16_N_2_O)_1_: C, 66.21; H, 9.53; N, 6.62; S, 5.05. Found: C, 58.70; H, 9.40; N, 6.98; S, 5.39.

#### 4.3.2. General synthetic procedure for citrate coordinated gold nanoparticles (according to the Turkevich method modified by Mattern *et al*. [5])

HAuCl_4_ · 3 H_2_O was dissolved in demineralized H_2_O and heated to reflux for 20 min. A solution of trisodium citrate dihydrate in demin. H_2_O was added quickly under vigorous stirring. The mixture was heated to 80°C for 2 h before cooling in an ice bath and filtrated using syringe filtration on a cellulose membrane with 0.2 µm pore size.

#### 4.3.3. Synthesis of Au-citrate (Ø 11 nm) in H_2_O

HAuCl_4_ · 3 H_2_O (50 mg, 0.13 mmol, 1.0 eq.) in demin. H_2_O (195 ml), trisodium citrate dihydrate (600 mg, 2.04 mmol, 15 eq.) in demin. H_2_O (5 ml). A pink, clear NP dispersion with a particle concentration of 14.6 nM was obtained and stored at 4°C in the dark.

**^1^H-NMR** (600 MHz, D_2_O): *δ*/ppm = 2.71–2,69 (m, 2H, C*H*_2_), 2.61–2,58 (m, 2H, C*H*_2_); **IR** (ATR): *ν*/cm^−1^ = 3425 (*ν*_O–H_), 1595 (*ν*_C = O_), 1249, 620; **TEM**: *d* = 11.2 ± 0.9 nm; **UV/Vis**: *λ*_max_ = 523 nm; **DLS**: *d*_hydr_ = 13 ± 5 nm.

#### 4.3.4. Synthesis of Au-citrate (Ø 12 nm) in H_2_O

HAuCl_4_ · 3 H_2_O (50 mg, 0.13 mmol, 1.0 eq.) in demin. H_2_O (195 ml), trisodium citrate dihydrate (400 mg, 1.36 mmol, 10 eq.) in demin. H_2_O (5 ml). A dark red, clear NP dispersion with a particle concentration of 11.9 nM was obtained and stored at 4°C in the dark. **TEM**: *d* = 12.0 ± 0.8 nm; **UV/Vis**: *λ*_max_ = 523 nm; **DLS**: *d*_hydr_ = 12 ± 7 nm.

#### 4.3.5. Synthesis of Au-citrate (Ø 13 nm) in H_2_O

HAuCl_4_ · 3 H_2_O (50 mg, 0.13 mmol, 1.0 eq.) in demin. H_2_O (195 ml), trisodium citrate dihydrate (254 mg, 0.87 mmol, 6.7 eq.) in demin. H_2_O (5 ml). A dark red, clear NP dispersion with a particle concentration of 9.7 nM was obtained and stored at 4°C in the dark. **TEM**: *d* = 12.8 ± 1.3 nm; **UV/Vis**: *λ*_max_ = 522 nm; **DLS**: *d*_hydr_ = 13 ± 3 nm.

#### 4.3.6. Synthesis of Au-citrate (Ø 14 nm) in H_2_O

HAuCl_4_ · 3 H_2_O (50 mg, 0.13 mmol, 1.0 eq.) in demin. H_2_O (195 ml), trisodium citrate dihydrate (224 mg, 0.76 mmol, 5.8 eq.) in demin. H_2_O (5 ml). A dark red, clear NP dispersion with a particle concentration of 7.8 nM was obtained and stored at 4°C in the dark. **TEM**: *d* = 13.8 ± 1.2 nm; **UV/Vis**: *λ*_max_ = 521 nm; **DLS**: *d*_hydr_ = 15 ± 3 nm.

#### 4.3.7. Synthesis of Au-citrate (Ø 16 nm) in H_2_O

HAuCl_4_ · 3 H_2_O (12.8 mg, 0.03 mmol, 1.0 eq.) in demin. H_2_O (47 ml), trisodium citrate dihydrate (38 mg, 0.13 mmol, 4.0 eq.) in demin. H_2_O (3 ml). A dark red, clear NP dispersion with a particle concentration of 5.1 nM was obtained and stored at 4°C in the dark. **TEM**: *d* = 15.9 ± 1.1 nm; **UV/Vis**: *λ*_max_ = 521 nm; **DLS**: *d*_hydr_ = 17 ± 4 nm.

### 4.4. General synthetic procedure for mercaptoundecanoic acid (MUDA) coordinated gold nanoparticles in H_2_O (according to the Stucky method modified by Mattern *et al.* [8])

PPh_3_AuCl was dissolved in DMSO and a solution of ligand dissolved in DMSO was added. The mixture was heated to 65°C and a solution of *^t^*Bu-amine borane complex in DMSO or solid *^t^*Bu-amine borane complex was added quickly under vigorous stirring. The dark red dispersion was stirred at 65°C for 3.5 h in the dark and cooled in an ice bath. Subsequently, the particles were precipitated with EtOH (10–14 ml) and centrifuged (45 min, 7000 r.p.m.). The supernatant was discarded, the dark residue was redispersed three times and washed again with EtOH. The obtained dark solid was dried in air and redispersed in H_2_O. Diluted NaOH (0.1–0.2 ml) was added to obtain a stable NP dispersion. Then, the dispersion was further purified via dialysis against H_2_O.

#### 4.4.1. Direct synthesis of Au-MUDA (Ø 8 nm) in H_2_O

PPh_3_AuCl (16 mg, 31 µmol, 1.0 eq.) in DMSO (3 ml), MUDA (30 mg, 138 µmol, 4.6 eq.) in DMSO (1 ml), *^t^*Bu-amine borane complex (27 mg, 300 µmol, 10 eq.) in DMSO (1 ml). The dark solid was redispersed in H_2_O (10 ml). Diluted NaOH (2 drops) was added to obtain a stable NP dispersion, which was further purified via dialysis against demin. H_2_O (in MWCO 12 000, 24 h). The violet, clear NP dispersion with a particle concentration of 212 nM was stored at 4°C in the dark.

**^1^H-NMR** (600 MHz, D_2_O): *δ*/ppm = 2.93–2.86 (m, 2 H, C*H*_2_), 2.17 (t, *J* = 6.6 Hz, 2 H, C*H*_2_), 1.75–1.68 (m, 2 H, C*H*_2_), 1.58–1.50 (m, 2 H, C*H*_2_), 1.45–1.36 (m, 2 H, C*H*_2_), 1.36–1.24 (m, 10 H, C*H*_2_); **IR** (ATR): *ν*/cm^−1^ = ca. 3300 (*ν*_O–H_, H_2_O), 2921 (*ν*_C–H_), 2846 (*ν*_C–H_), 1675, 1555 (*ν*_C = O_), 1533, 1443, 1406 (*δ*_C–H_), 1293 (*ν*_C–O_), 955, 723 (*δ*_C–H_); **TEM**: *d* = 7.9 ± 0.9 nm; **UV/Vis**: *λ*_max_ = 527 nm; **DLS**: *d*_hydr_ = 12 ± 3 nm.

#### 4.4.2. Direct synthesis of Au-MUDA (Ø 9 nm) in H_2_O

PPh_3_AuCl (16 mg, 31 µmol, 1.0 eq.) in DMSO (3 ml), MUDA (7 mg, 30 µmol, 1.0 eq.) in DMSO (1 ml), *^t^*Bu-amine borane complex (27 mg, 300 µmol, 10 eq.) in DMSO (1 ml). The dark solid was redispersed in H_2_O (10 ml). Diluted NaOH (3 drops) was added to obtain a stable NP dispersion, which was further purified via dialysis against demin. H_2_O (in MWCO 12 000, 24 h). The violet, clear NP dispersion with a particle concentration of 143 nM was stored at 4°C in the dark. **TEM**: *d* = 9.0 ± 0.9 nm; **UV/Vis**: *λ*_max_ = 528 nm; **DLS**: *d*_hydr_ = 14 ± 4 nm.

#### 4.4.3. Direct synthesis of Au-MUDA (Ø 10 nm) in H_2_O

PPh_3_AuCl (16 mg, 31 µmol, 1.0 eq.) in DMSO (3 ml), MUDA (5 mg, 20 µmol, 0.7 eq.) in DMSO (1 ml), *^t^*Bu-amine borane complex (2 mg, 20 µmol, 0.7 eq.) in DMSO (1 ml). The dark solid was redispersed in H_2_O (10 ml). Diluted NaOH (3 drops) was added to obtain a stable NP dispersion, which was further purified via dialysis against demin. H_2_O (in MWCO 12 000, 24 h). The violet, clear NP dispersion with a particle concentration of 115 nM was stored at 4°C in the dark. **TEM**: *d* = 9.7 ± 1.0 nm; **UV/Vis**: *λ*_max_ = 525 nm; **DLS**: *d*_hydr_ = 12 ± 3 nm.

### 4.5. Syntheses of atropine-functionalized gold nanoparticles

#### 4.5.1. Synthesis of Au-MUDA-AT (Ø 8 nm) in H_2_O

Au-MUDA NPs with Ø 10 nm (3.5 ml) diluted in demin. H_2_O (6 ml), MUDA-AT (35 mg, 72 µmol) in DMSO (0.5 ml), NEt_3_ (3 drops). The NP dispersion was purified via dialysis against demin. H_2_O (in MWCO 12 000, 5 × 2 h). A pink, clear NP dispersion with a concentration of 139 nM was obtained and stored at 4°C in the dark.

**^1^H-NMR** (600 MHz, D_2_O): *δ*/ppm = 7.43–7.19 (m, 5 H, C*H*), 5.45 (s, 1 H, C*H*), 4.47–4.30 (m, 1 H, C*H*), 4.00–3.84 (m, 1 H, C*H*), 3.80–3.75 (m, 1 H, C*H*), 3.17–2.89 (m, 2 H, C*H*_2_), 2.56 (s, 3 H, C*H*_3_), 2.46–2.38 (m, 4 H, C*H*_2_), 2.27–1.99 (m, 8 H, C*H*_2_), 1.63–1.49 (m, 6 H, C*H*_2_), 1.42–1.15 (m, 10 H, C*H*_2_); **IR** (ATR): *ν*/cm^−1^ = 3308 (*ν*_O–H_), 2921, 2841 (*ν*_C–H_), 1695 (*ν*_C=O_), 1646 (*ν*_C=O_), 1557 (*ν*_C=C_), 1448, 1417 (*δ*_C–H_), 1250 (*δ*_C–O_), 1184 (*δ*_C–O_), 1029 (*ν*_C–N_), 813 (*δ*_C–H_), 729 (*ν*_C–S_), 700 (*δ*_C–H_); **TEM**: *d* = 8.0 ± 0.8 nm; **UV/Vis**: *λ*_max_ = 530 nm; **DLS**: *d*_hydr_ = 29 ± 8 nm.

#### 4.5.2. Synthesis of Au-MUDA-AT (Ø 9 nm) in H_2_O

Au-MUDA NPs with Ø 9 nm (3.5 ml) diluted in demin. H_2_0 (5.5 ml), solid MUDA-AT (34 mg, 69 µmol), diluted NaOH (2 drops). The nanoparticle solution was purified via dialysis against demin. H_2_O (in MWCO 12 000, 26 h). A pink, clear NP solution with a concentration of 97 nM was obtained and stored at 4°C in the dark. **TEM**: *d* = 9.1 ± 0.7 nm; **UV/Vis**: *λ*_max_ = 530 nm; **DLS**: *d*_hydr_ = 15 ± 6 nm.

#### 4.5.3. Synthesis of Au-MUDA-AT (Ø 10 nm) in H_2_O

Au-MUDA NPs with Ø 10 nm (1 ml) diluted in demin. H_2_O (9 ml), MUDA-AT (25 mg, 36 µmol) in DMSO (0.4 ml), NEt_3_ (3 drops). After degassing of the NP dispersion, MUDA-AT (15 mg in 0.2 ml DMSO) was slowly added. To ensure a stable dispersion, NEt_3_ (3 drops) was added and the reaction mixture was stirred at room temperature for 45 min. Further MUDA-AT (10 mg in 0.2 ml DMSO) was added dropwise and the mixture was stirred at room temperature for 72 h. Then, the NP dispersion was purified via dialysis against demin. H_2_O (in MWCO 6000, 6 × 2 h). A pink, clear NP dispersion with a concentration of 19.0 M was obtained and stored at 4°C in the dark. **TEM**: *d* = 9.7 ± 0.9 nm; **UV/Vis**: *λ*_max_ = 530 nm; **DLS**: *d*_hydr_ = 33 ± 4 nm.

#### 4.5.4. Synthesis of Au-MUDA-AT (Ø 11 nm) in H_2_O

Au-citrate NPs with Ø 11 nm (10 ml), MUDA-AT (25 mg, 36 µmol) in DMSO (0.4 ml), NEt_3_ (3 drops). After degassing of the NP dispersion, MUDA-AT (15 mg in 0.2 ml DMSO) was slowly added. To ensure a stable dispersion NEt_3_ (3 drops) was added and the reaction mixture was stirred at room temperature for 45 min. Further MUDA-AT (10 mg in 0.2 ml DMSO) was added dropwise and stirred at room temperature for 72 h. Then, the NP dispersion was purified via dialysis against demin. H_2_O (in MWCO 6000, 6 × 2 h). A pink, clear NP dispersion with a concentration of 15.2 nM was obtained and stored at 4°C in the dark. **TEM**: *d* = 11.2 ± 1.1 nm; **UV/Vis**: *λ*_max_ = 523 nm; **DLS**: *d*_hydr_ = 31 ± 5 nm.

#### 4.5.5. Synthesis of Au-MUDA-AT (Ø 12 nm) in H_2_O

Au-citrate NPs with Ø 12 nm (10 ml), MUDA-AT (40 mg, 82 µmol) in DMSO (0.2 ml), diluted NaOH (5 drops). The NP dispersion was purified via dialysis against demin. H_2_O (in MWCO 12 000, 3 × 2 h). A pink, clear NP dispersion with a concentration of 11 nM was obtained and stored at 4°C in the dark. **TEM**: *d* = 12.0 ± 1.0 nm; **UV/Vis**: *λ*_max_ = 529 nm; **DLS**: *d*_hydr_ = 112 ± 59 nm.

#### 4.5.6. Synthesis of Au-MUDA-AT (Ø 13 nm) in H_2_O

Au-citrate NPs with Ø 13 nm (10 ml), MUDA-AT (30 mg, 61 µmol) in DMSO (0.2 ml), NEt_3_ (5 drops). The NP dispersion was purified via dialysis against demin. H_2_O (in MWCO 12 000, 9 × 2 h). A pink, clear NP dispersion with a concentration of 8.7 nM was obtained and stored at 4°C in the dark. **TEM**: *d* = 12.9 ± 1.0 nm; **UV/Vis**: *λ*_max_ = 528 nm; **DLS**: *d*_hydr_ = 105 ± 42 nm.

#### 4.5.7. Synthesis of Au-MUDA-AT (Ø 14 nm) in H_2_O

Au-citrate NPs with Ø 14 nm (10 ml), MUDA-AT (30 mg, 61 µmol) in DMSO (0.2 ml), NEt_3_ (5 drops). The NP dispersion was purified via dialysis against demin. H_2_O (in MWCO 6000, 9 × 2 h). A pink, clear NP dispersion with a concentration of 7.5 nM was obtained and stored at 4°C in the dark. **TEM**: *d* = 14.0 ± 1.0 nm; **UV/Vis**: *λ*_max_ = 527 nm; **DLS**: *d*_hydr_ = 31 ± 13 nm.

#### 4.5.8. Synthesis of Au-MUDA-AT (Ø 16 nm) in H_2_O

Au-citrate NPs with Ø 16 nm (10 ml), MUDA-AT (30 mg, 61 µmol) in DMSO (0.5 ml), NEt_3_ (3 drops). The NP dispersion was purified via dialysis against demin. H_2_O (in MWCO 6000, 5 × 2 h). A pink, clear NP dispersion with a concentration of 3.6 nM was obtained and stored at 4°C in the dark. **TEM**: *d* = 15.9 ± 1.1 nm; **UV/Vis**: *λ*_max_ = 528 nm; **DLS**: *d*_hydr_ = 53 ± 19 nm.

### 4.6. Animals

Female and male Wistar rats with a body mass of 200–250 g were used for the Ussing chamber and organ bath experiments. The animals were bred and housed at the Institute for Veterinary Physiology and Biochemistry of the Justus Liebig University Giessen at an ambient temperature of 22.5°C and air humidity of 50–55% on a 12 h : 12 h light-dark cycle with free access to water and food until the time of the experiment.

### 4.7. Solutions

The standard solution for the Ussing chamber experiments was a buffer solution containing (mmol l^−1^): NaCl 107, KCl 4.5, NaHCO_3_ 25, Na_2_HPO_4_ 1.8, NaH_2_PO_4_ 0.2, CaCl_2_ 1.25, MgSO_4_ 1 and glucose 12. The solution was gassed with carbogen (5% CO_2_ in 95% O_2_, v/v); pH was 7.4.

### 4.8. Tissue preparation

Animals were killed in CO_2_ narcosis by cervical dislocation followed by exsanguination. The jejunum was extracted and washed with ice-cold buffer solution. For Ussing chamber experiments, the jejunum was placed on a small plastic rod and opened longitudinally before it was fixed between the Ussing half chambers. For the contractility studies with serosal drug application, the jejunum was fixed on a small plastic rod and 2 cm long segments were ligated before they were transferred into organ bath chambers to measure isometric contractions of the longitudinal muscle layer. For isometric force measurement after mucosal drug administration, the jejunum was placed on a small plastic rod with a notch on one end. A ligature was set in the notch to fix the jejunum on the rod and the intestine was carefully inverted so that the mucosa formed the outer surface of the resulting tube. After closing the lower end of the inverted tube with a ligature, the length of the jejunal segments was standardized to 10 cm. Each 10 cm segment was filled with 2.5 ml ice-cold buffer solution. Finally, segments of 2 cm length were ligated from the 10 cm tube and incubated in the organ bath chambers. For each experiment, two segments of the jejunum of each rat were prepared.

### 4.9. Short-circuit current measurements

The mucosa-submucosa preparation was fixed in a modified Ussing chamber bathed with a volume of 3.5 ml on each side of the jejunal tissue. The tissue was incubated at 37°C and short-circuited by a computer-controlled voltage-clamp device (Ingenieur Büro für Mess- und Datentechnik Mussler, Aachen, Germany) with correction for solution resistance. Short-circuit current (*I*_sc_) was continuously recorded on a chart-recorder. *I*_sc_ is expressed as µEq h^−1^ cm^−2^, i.e. the flux of a monovalent ion per time and area, with 1 µEq h^−1^ cm^−2^ = 26.9 µA cm^−2^. Tissue conductance (*G*_t_; in mS cm^−2^) was measured every minute by the voltage deviation induced by a current pulse (± 50 µA, duration 200 ms) under open-circuit conditions. Baseline of *I*_sc_ and *G*_t_ were calculated as an average of *I*_sc_ and *G*_t_ over 3 min prior to each drug application. The maximum increase or decrease of *I*_sc_ and *G*_t_ was measured as difference to the baseline during 10 min after drug application.

### 4.10. Isometric force measurements and electrical field stimulation

Change of force (in gram) generated by the jejunal segments due to relaxation and contraction of intestinal smooth muscle cells in the longitudinal layer of the tunica muscularis was recorded under isometric conditions. The organ bath chamber was filled with carbogen-gassed buffer solution, constantly warmed up to 37°C. First, a pre-tension of 2 g was applied, then the tension was manually reduced to 1 g. Subsequently, the following protocols were used for isometric force measurement after serosal (1) and mucosal (2) drug application using either non-inverted (1) or inverted segments of small intestine (2):

(1) After an equilibrium period of at least 15 min, Au-MUDA-AT NPs or equivalent volumes of buffer were applied followed by the administration of CCh (5 × 10^−7^ mol l^−1^ at the serosal side) 20 min later. KCl (15 × 10^−3^ mol l^−1^ at the serosal side) served as viability control at the end of each experiment. The baseline prior to drug application was calculated over 1 min, while the maximum increase of isometric force was calculated as difference to the baseline within a period of 3 min after drug application.
(2) After an equilibrium period of 20 min, the inverted tissues were stimulated with an electrical field which was applied by two electrodes. Electrical stimuli were applied with an electric current of 250 mA, a pulse duration of 1 ms over 10 s, a frequency of 10 Hz and were repeated every 2 min. After 30 min of electrical stimulation, the first drug was applied. The baseline was calculated as average of the isometric tension over 10 s prior to each electrical stimulation. EFS-induced change of force was calculated as the difference between the maximum increase of isometric tension within 30 s after EFS and the baseline.

### 4.11. Drugs

Atropine, Au-MUDA-AT, carbachol, potassium chloride (Mettler Toledo, Greifensee, Switzerland) were dissolved in aqueous stock solutions; forskolin (Tocris, Bristol, UK) and indomethacin were dissolved in ethanol. If not indicated otherwise, drugs were from Sigma (Steinheim, Germany).

### 4.12. Statistics

Results are given as mean ± standard error of the mean (s.e.m.) with the number (*n*) of investigated tissues. For the comparison of two groups either Student's *t*-test or Mann–Whitney *U*-test was applied. An *F*-test decided which test method had to be used. When more than two groups had to be compared, an analysis of variances (ANOVA) was performed. If an *F*-test indicated that variances between the groups were significantly larger than within the groups, Tukey *post hoc* test was performed. *p* < 0.05 was considered to be statistically significant.

## Data Availability

Supporting information concerning synthesis and analysis of the nanoparticles and isometric force data referred to as ‘data not shown’ are deposited at Dryad Digital Repository: https://doi.org/10.5061/dryad.dz08kps05 [57].
